# A real-world disproportionality analysis of roflumilast using the US food and drug administration adverse event reporting system data

**DOI:** 10.3389/fdsfr.2025.1721656

**Published:** 2025-12-12

**Authors:** Baiqing Huang, Yirou Ma, Wenli Wang, Shaorui Gu, Xisheng Wang

**Affiliations:** Department of Cardiothoracic Surgery, Tongji Hospital, School of Medicine, Tongji University, Shanghai, China

**Keywords:** roflumilast, adverse drug reactions, pharmacovigilance, FDA adverse event reporting system (FAERS), real-world evidence

## Abstract

**Introduction:**

Roflumilast, a selective phosphodiesterase-4 inhibitor, is prescribed to reduce exacerbations in severe COPD, but its real-world safety profile remains insufficiently characterized.

**Methods:**

We conducted a retrospective pharmacovigilance study using the FDA Adverse Event Reporting System (FAERS). Reports from 2004 to 2025Q1 listing roflumilast as the primary suspect were extracted, deduplicated, and analyzed. Disproportionality analysis employed four algorithms—Reporting Odds Ratio (ROR), Proportional Reporting Ratio (PRR), Bayesian Confidence Propagation Neural Network (BCPNN), and Multi-item Gamma Poisson Shrinker (MGPS). Safety signals were defined by established thresholds. Time-to-onset and Weibull modeling were applied to assess temporal patterns.

**Results:**

A total of 3,140 reports were identified, primarily involving older COPD patients. The median time-to-onset was 4 days (IQR 0–34), with 72% occurring within 30 days. Thirty Preferred Terms met signal criteria. Frequent signals included diarrhoea, weight decreased, nausea, dyspnoea, insomnia, and headache. Psychiatric events such as depression and suicidal ideation were notable. The strongest disproportionality was observed for gastroduodenal ulcer (ROR 47.1) and COPD (ROR 25.7). System Organ Class enrichment was most evident in gastrointestinal, psychiatric, and respiratory disorders.

**Discussion/Conclusion:**

This real-world analysis confirms roflumilast’s established adverse effects (gastrointestinal upset, weight loss, insomnia) and highlights concerning psychiatric signals. Most events occurred early, underscoring the need for close monitoring during treatment initiation. The use of multiple disproportionality methods enhances signal detection robustness and supports ongoing pharmacovigilance in clinical practice.

## 1 Introduction

Chronic Obstructive Pulmonary Disease (COPD) is a common chronic condition characterized by airflow obstruction, encompassing chronic bronchitis and/or emphysema, which can progress to cor pulmonale and respiratory failure. It stands as a leading global cause of morbidity, mortality, and healthcare utilization (Chr et al., 2022). Roflumilast is a selective phosphodiesterase-4 (PDE4) inhibitor approved for reducing acute exacerbations in patients with severe COPD, and its emulsion formulation was approved this year for the treatment of psoriasis (Fabbri et al., 2010; Cordoro, 2022). In large clinical trials, Roflumilast improved lung function and quality of life but was also associated with adverse drug reactions (ADRs) such as gastrointestinal adverse reactions, weight loss, and psychiatric symptoms (insomnia, anxiety, depression) (Janjua et al., 2020). Notably, rare cases of suicidal ideation have been reported early in therapy (Oba and Lone, 2013). Post-marketing surveillance is therefore important to characterize Roflumilast’s real-world safety profile.

The FDA Adverse Event Reporting System (FAERS) constitutes a publicly accessible database that aggregates spontaneous adverse event (AE) reports submitted by healthcare professionals, manufacturers, and consumers. FAERS represents the largest global pharmacovigilance repository and is extensively utilized for safety signal detection. Disproportionality analyses within FAERS commonly utilize several statistical indices—including the Reporting Odds Ratio (ROR), Proportional Reporting Ratio (PRR), Bayesian Confidence Propagation Neural Network (BCPNN), and Multi-item Gamma Poisson Shrinker (MGPS)—to detect disproportionately reported drug–AE pairs (Zhao et al., 2024). Previous research has applied these methodologies to identify safety signals for various pharmaceuticals, such as mesalazine, durvalumab, among others (Zhang et al., 2024; Tan et al., 2025). Signal detection is enhanced in robustness when multiple algorithms demonstrate concordance, as each method possesses distinct strengths and inherent biases.

Quantitative assessments of reports were conducted using signal assays, which identify drug-related adverse events. FAERS has been utilized to analyze various drugs and diseases to detect potential adverse events requiring special attention, thereby guiding clinical use and treatment (Zhao et al., 2024).

This study performs a comprehensive pharmacovigilance analysis of Roflumilast using FAERS data mining. We first characterize the profiles of Roflumilast reports, including demographics, indications, outcomes, and time-to-onset. Subsequently, we apply four disproportionality analysis algorithms—ROR, PRR, BCPNN, and MGPS—employing standard threshold criteria to detect significant ADRs. To facilitate interpretation, we investigate the organ-system distribution of detected signals and utilize Weibull shape parameter modeling to analyze AE onset patterns. The objective is to deliver a transparent and data-driven assessment of Roflumilast’s safety profile in real-world clinical practice.

## 2 Methods

### 2.1 Data source and case identification

**FIGURE 1:**
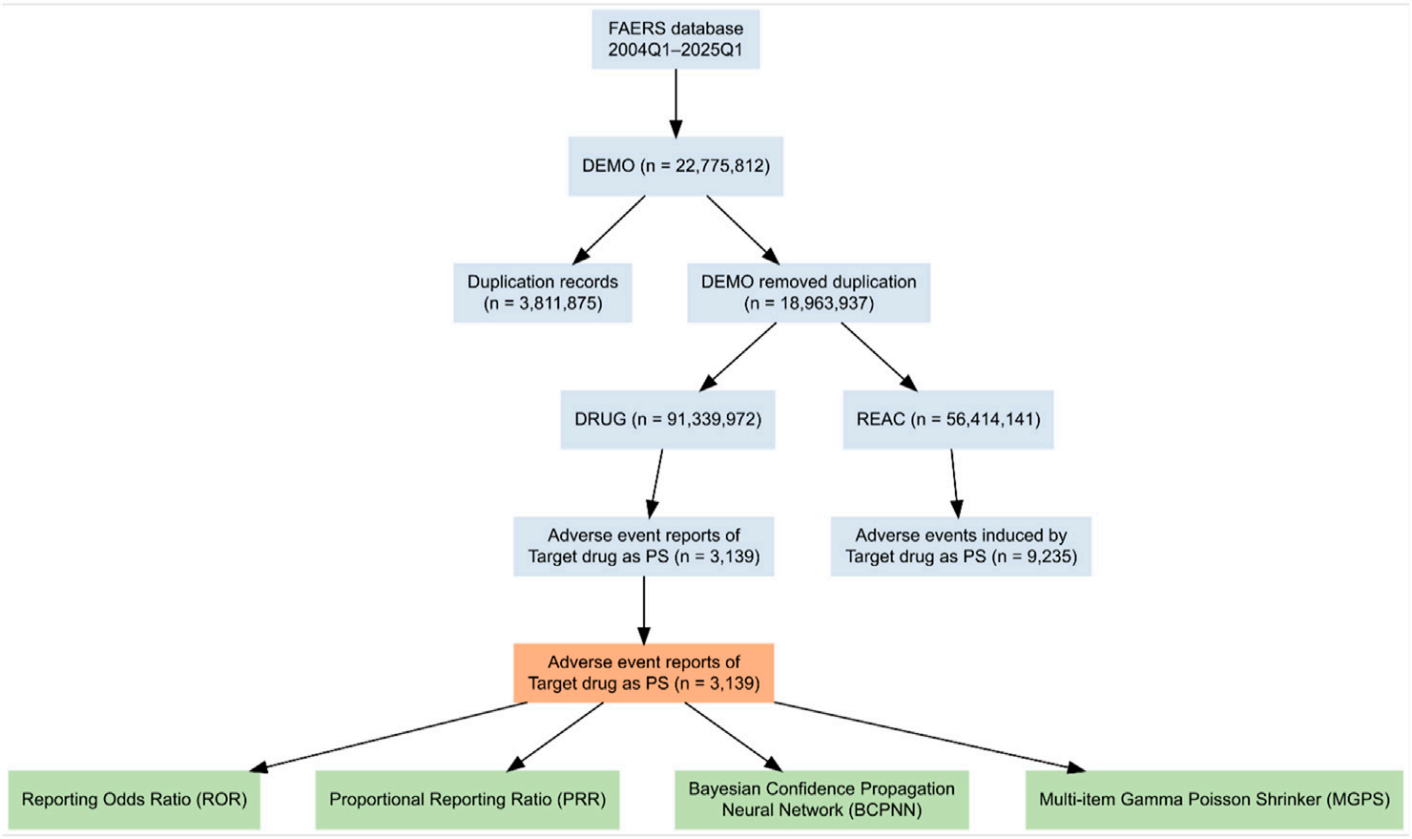
Overview of FAERS data structure and preprocessing workflow. DEMO, DRUG, and REAC represent the demographic, drug, and reaction datasets, respectively. Duplicated case records were removed before merging DRUG and REAC tables for analysis. Abbreviations: FAERS, Food and Drug Administration Adverse Event Reporting System; DEMO, demographic file; DRUG, drug information file; REAC, reaction information file; PS, primary suspect.

The FAERS database consists of four main files: DEMO (demographic information), DRUG (drug information), REAC (reaction information), and THER (therapy information). Each quarterly dataset (Q1, Q2, etc.) is coded according to MedDRA (Medical Dictionary for Regulatory Activities). FAERS data files (including DEMO, DRUG, REAC, and THER) covering reports submitted from Q1 2004 through Q1 2025 were downloaded from the FDA website. Standardized deduplication was performed by retaining the most recent FDA receipt date and latest report version for each unique case identifier. Subsequently, all case reports designating Roflumilast (including the brand name “Daliresp” and synonymous designations) as a Primary Suspect (PS) medication were selected. Reports were excluded if critical data fields (suspect drug information, adverse event date) were incomplete or invalid. All AEs information was systematically coded using the MedDRA hierarchical structure, encompassing System Organ Class (SOC) and Preferred Term (PT) levels. The overall data processing steps are illustrated in Figure 1. After deduplication, the DRUG and REAC tables were linked through the unique case identifier to identify reports listing roflumilast as the primary suspect (PS) drug, forming the final analysis dataset.

### 2.2 Descriptive analysis

Patient and report characteristics (including age, sex, weight, reporter type, country, indication, outcomes, and therapy dates) were summarized using frequencies and proportions. The primary indication for Roflumilast therapy was documented. For time-to-onset analysis, the interval between EVENT_DT (event onset date) and START_DT (treatment initiation date) was calculated. Reports exhibiting inconsistent temporal data (onset date preceding treatment initiation) were excluded from onset latency analysis. Serious outcomes (including death, life-threatening events, hospitalization, and disability) were recorded.

### 2.3 Signal detection

Disproportionality analyses utilizing case/non-case methodology were performed to identify potential safety signals associated with Roflumilast. Four established quantitative signal detection algorithms were employed: the ROR the PRR, the BCPNN (information component metric, IC025), and the MGPS (Empirical Bayes Geometric Mean metric, EBGM05). Calculations were performed using 2 × 2 contingency tables, where ‘a’ represents the number of reports listing Roflumilast and the specific AE, ‘b’ denotes reports listing Roflumilast with other AEs, ‘c’ signifies reports listing other drugs with the specific AE, and ‘d’ represents reports listing other drugs with other AEs. A positive safety signal was defined according to the following criteria, consistent with published standards: a minimum of three reports (*N*≥3); ROR lower 95% confidence interval (CI) > 1; PRR ≥2 with an associated χ^2^ statistic ≥4; IC025 > 0; and EBGM05 > 2. These thresholds represent standard criteria for robust signal detection. Preferred Terms (PTs) meeting all four conditions concurrently were identified. Results are presented as ROR (95% CI), PRR (χ^2^), IC025, and EBGM05 for each identified signal.

### 2.4 Organ/system enrichment analysis

To determine which organ systems were predominantly involved, signals were aggregated by SOC. For each SOC, all Roflumilast-related PTs within that classification were consolidated, and a composite disproportionality measure was computed using aggregated case counts. This SOC-level ROR and its associated metrics quantify system-wide signal enrichment.

### 2.5 Time-to-onset analysis

The distribution of time-to-onset (TTO) for Roflumilast AEs was characterized by median and interquartile range (IQR). We further applied Weibull distribution modeling to the TTO data. The Weibull shape parameter β indicates the failure type: β < 1 suggests an early-failure (events clustered early after initiation), β ≈ 1 indicates a random occurrence, and β > 1 indicates a wear-out pattern (risk increases over time). We estimated Weibull parameters (scale α and shape β with 95%CI) using established statistical software. A complementary Kaplan–Meier plot could be generated but was not included in the manuscript due to overlapping information with the Weibull analysis.

### 2.6 Statistics and software

All statistical analyses were performed using R version 4.4.2. Significance thresholds for signal detection adhered to the criteria specified previously. The calculation method is shown in Tables 1,2. Beyond the calculation of disproportionality metrics, no inferential statistical comparisons (e.g., p-values) were conducted. As this study utilized de-identified, publicly available data, formal ethics approval was not required.

**TABLE 1:** Summary of major algorithms used for signal detection.

Algorithms	Indicator	Equation	Criteria
ROR	ROR	ROR = (ad)/(bc)	ROR05 > 1, N ≥ 2
		95%CI = e^ln(ROR)±1.96(1/a+1/b+1/c+1/d)^0.5^	
PRR	PRR	PRR = [a/(a+b)]/[c/(c + d)]	PRR≥2
	χ^2^	χ^2^ = [(ad-bc)^2^ (a+b + c + d)]/[(a+b)(c + d)(a+c)(b + d)]	χ^2^ ≥ 4, N ≥ 3
BCPNN	IC	IC = log_2_ [a (a+b + c + d)]/[(a+c)(a+b)]	IC025 > 0
		95%CI = e^ln(IC)±1.96(1/a+1/b+1/c+1/d)^0.5^	
MGPS	EBGM	EBGM = a (a+b + c + d)/(a+c)/(a+b)	EBGM05 > 2, N ≥ 0
		95%CI = e^ln(EBGM)±1.96(1/a+1/b+1/c+1/d)^0.5^	

Abbreviations: ROR, reporting odds ratio; PRR, proportional reporting ratio; BCPNN, Bayesian confidence propagation neural network; MGPS, multi-item gamma Poisson shrinker; CI, Confidence Interval; IC025, lower 95% credibility limit of Information Component; EBGM05, Empirical Bayes Geometric Mean, lower 5% confidence limit.

**TABLE 2:** Two-by-two contingency table for disproportionality analyses.

	Target AEs	All other AEs	Total
Target drug	a	b	a+b
All other drugs	c	d	c + d
Total	a+c	b + d	a+b + c + d

Abbreviations: AE, adverse event.

## 3 Result

### 3.1 General characteristics

**FIGURE 2:**
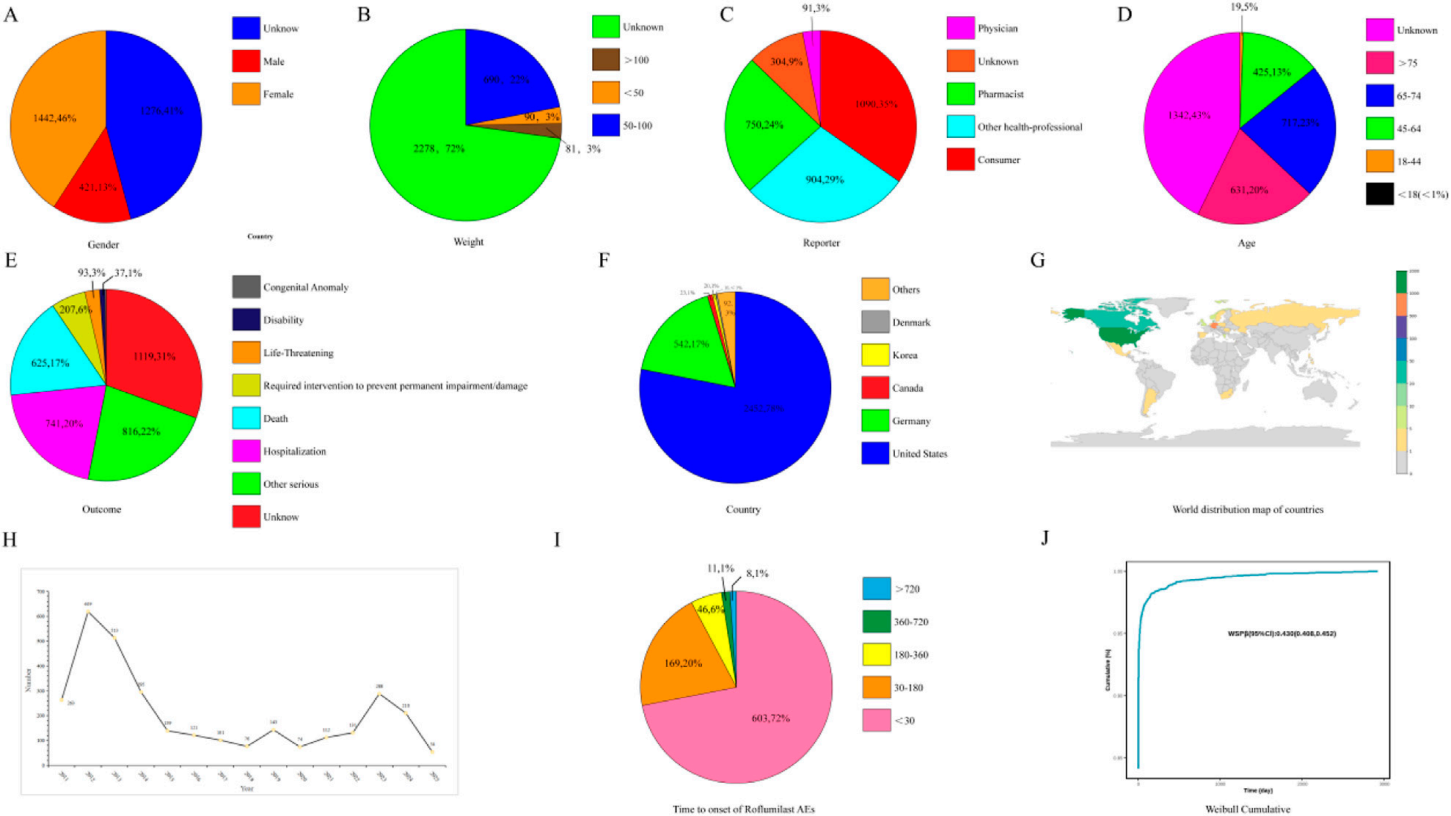
Clinical characteristics of reports with Roflumilast from the FAERS database. **(A)** The gender distribution of AEs of roflumilast. **(B)** The weight distribution of AEs of roflumilast. **(C)** A distribution chart of the identities of reporters of AEs of roflumilast. **(D)** A chart showing the age distribution of cases of AEs associated with roflumilast. **(E)** The distribution of outcomes for cases of AEs associated with roflumilast. **(F)** The distribution of countries reporting AEs associated with roflumilast. **(G)** World distribution map of countries reporting AEs to roflumilast. **(H)** The number of AEs reports each year. **(I)** Time to onset of Roflumilast AEs. **(J)** Weibull Cumulative Distribution of Time to Onset for Roflumilast Events. Abbreviations: PT, Preferred Term; AE, adverse event; ROR, reporting odds ratio.

A total of 3,140 unique Roflumilast-associated reports were identified in FAERS (Q1 2004–Q1 2025). Gender distribution was balanced (Table 3; Figure 2A). The weight distribution primarily ranges from 50 kg to 100 kg, with a minor proportion falling below 50 kg and above 100 kg (Table 3; Figure 2B). Primary reporters predominantly comprised consumers (34.7%) and physicians (23.9%), with pharmacists accounting for a minimal 2.9% (Table 3; Figure 2C). Most cases occurred in older patients: only 0.8% were aged <45 years, whereas approximately 23% were aged 65–75 years and 20% were aged >75 years (Table 3; Figure 2D). The primary indication was chronic obstructive pulmonary disease. The next most common indication was psoriasis (Table 3). Serious outcomes were documented in a substantial minority of cases: death occurred in 17.2%, hospitalization in 20.4%, life-threatening events in 2.6%, and disability in 1.0%. Furthermore, an additional 5.7% necessitated intervention to avert permanent damage). Cumulatively, serious outcomes (encompassing life-threatening events, hospitalization, disability, or death) occurred in approximately 41% of cases. These findings underscore that while most reports involved patients diagnosed with COPD, a substantial proportion experienced notable adverse outcomes (Table 3; Figure 2E). Geographically, reports were heavily concentrated in North America and Europe: the United States accounted for 78.1% of reports, followed by Germany (17.3%), with negligible contributions from other countries (Table 3; Figure 2F,G). Moreover, the number of reported cases has shown signs of resurgence in recent years (Figure 2H).

**TABLE 3:** Clinical characteristics of reports with Roflumilast from the FAERS database.

Item	n (%)
Gender	
Male	1,442 (45.94)
Female	1,276 (40.65)
Unknow	421 (13.41)
Age	
<18	5 (0.16)
18–44	19 (0.61)
45–64	425 (13.54)
65–75	717 (22.84)
>75	631 (20.10)
UnKnow	1,342 (42.75)
Weight	
<50 kg	90 (2.87)
50 kg–100 kg	690 (21.98)
>100 kg	81 (2.58)
UnKnow	2,278 (72.57)
Reporter	
Consumer	1,090 (34.72)
Physician	750 (23.89)
Pharmacist	91 (2.9)
Other	904 (28.80)
UnKnow	304 (9.68)
Country top 5	
United States	2,452 (78.11)
Germany	542 (17.27)
Canada	23 (0.73)
Korea (South)	20 (0.64)
Denmark	10 (0.32)
Outcome	
Other	816 (22.42%)
Hospitalization	741 (20.36%)
Life-threatening	93 (2.56%)
Disability	37 (1.02%)
Death	625 (17.18%)
Unknown	1,119 (30.75%)
Congenital anomaly	1 (0.03%)
Required intervention to prevent permanent impairment/damage	207 (5.69%)
Indication top 5	
Chronic obstructive pulmonary disease	1,523 (75.06)
Psoriasis	107 (5.27)
Emphysema	36 (1.77)
Asthma	33 (1.63)
Seborrhoeic dermatitis	33 (1.63)
Onset time (days)	
<30	603 (72.04)
30–180	169 (20.19)
180–360	46 (5.5)
360–720	11 (1.31)
>720	8 (0.96)
Median (Q1, Q3)	4 (0, 34)
Year	As shown in Figure 2H

### 3.2 Time to onset of roflumilast-associated adverse events

TTO data were documented for 837 reports. Roflumilast-associated AEs demonstrated rapid onset, with a median TTO of 4 days post-treatment initiation (interquartile range [IQR] 0–34 days), and 72.0% of events occurring within 30 days (Figure 2J; Table 4). Weibull modeling estimated a shape parameter β ≈ 0.57 (<1), indicating an early-failure pattern characterized by peak risk proximate to treatment commencement. Consequently, the hazard rate for AEs declined temporally from initiation (Figure 2J). This temporal clustering suggests that most Roflumilast-related events manifest shortly after therapy initiation. Kaplan-Meier analysis of TTO exhibited a pronounced initial decline, corroborating the Weibull model results.

**TABLE 4:** Time-to-onset analysis of adverse events associated with roflumilast using weibull distribution modeling.

Drug	Time to onset (day)		Weibull distribution		
ROFLUMILAST	Case reports	Median (IQR)	Scale parameter:α(95%Cl)	Shape parameter:β(95%Cl)	Type
	837	4 (0–34)	0.573 (0.539–0.607)	38.653 (32.893–44.413)	Early failure

Abbreviations: AE, adverse event; IQR, interquartile range.

### 3.3 Adverse events by system organ class

**FIGURE 3:**
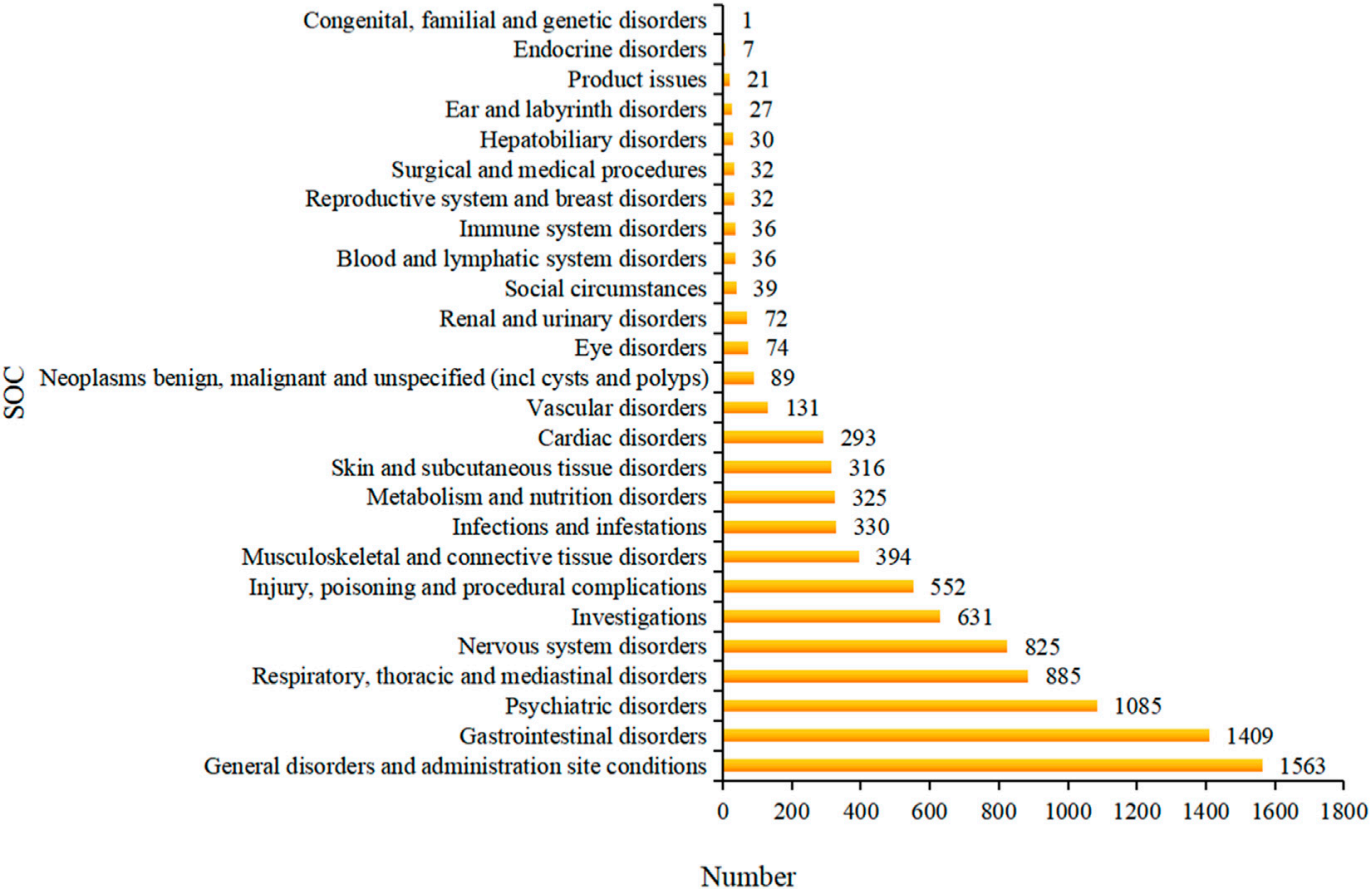
Bar chart showing the cumulative frequency of adverse event reports across SOCs based on MedDRA hierarchy. Abbreviations: SOC, system organ class; AE, adverse event; CI, confidence interval.

Adverse events were distributed across multiple SOCs, with certain domains showing disproportionately high cumulative counts. As illustrated in Figure 3, Gastrointestinal disorders and Psychiatric disorders were the most frequently reported categories, together accounting for more than one-third of all events. Other notable SOCs included General disorders, Nervous system disorders, and Respiratory disorders, consistent with the clinical pharmacology of Roflumilast. These frequency-based trends (Table 5) were further visualized in the bar chart, highlighting the relative burden across organ systems. The predominance of gastrointestinal and psychiatric events aligns closely with Roflumilast’s established safety profile.

**TABLE 5:** Distribution of adverse events involving organ systems under positive signals.

soc_name_en	Number	Proportion
Gastrointestinal disorders	808	19.57%
Psychiatric disorders	781	18.92%
General disorders and administration site conditions	586	14.20%
Nervous system disorders	565	13.69%
Respiratory, thoracic and mediastinal disorders	530	12.84%
Investigations	401	9.71%
Metabolism and nutrition disorders	230	5.57%
Musculoskeletal and connective tissue disorders	126	3.05%
Injury, poisoning and procedural complications	72	1.74%
Cardiac disorders	15	0.36%
Social circumstances	8	0.19%
Infections and infestations	3	0.07%
Neoplasms benign, malignant and unspecified (incl cysts and polyps)	3	0.07%

The number of cases represents the cumulative occurrences of adverse events under the System Organ Class (SOC). Proportion = number of risk signal cases under the System Organ Class (SOC)/total number of risk signal cases.

At the disproportionality level, certain SOCs showed clear enrichment. Psychiatric disorders and Respiratory, thoracic and mediastinal disorders exhibited the highest disproportionality signals, with aggregated RORs of 2.24 and 2.15, respectively (Table 6). By contrast, many other SOCs had ROR values <1.0, indicating no evidence of signal enrichment. Consistent with these findings, 25% of all significant PTs were classified under Psychiatric disorders, whereas 15.4% fell within Respiratory, thoracic and mediastinal disorders (Table 7). Additional SOCs with detectable enrichment included Investigations (11.5%), General disorders and administration site conditions (9.6%), Gastrointestinal disorders (7.7%), and Nervous system disorders (7.7%). Collectively, these organ-system patterns correspond well to the anticipated spectrum of adverse events related to PDE4 inhibition.

**TABLE 6:** Signal strength of SOCs involved in rofumilast-related PTs.

SOC	Number	ROR (95%CI)	PRR (X2)	IC025/IC-2SD	EBGM05
General disorders and administration site conditions	1,563	0.96 (0.91, 1.02)	0.97 (1.69)	−0.12	0.92
Gastrointestinal disorders	1,409	1.94 (1.83, 2.05)	1.8 (543.02)	0.76	1.7
Psychiatric disorders	1,085	2.24 (2.1, 2.39)	2.1 (658.24)	0.97	1.97
Respiratory, thoracic and mediastinal disorders	885	2.15 (2, 2.3)	2.04 (490.48)	0.92	1.9
Nervous system disorders	825	1.06 (0.99, 1.14)	1.06 (2.74)	−0.03	0.98
Investigations	631	1.12 (1.04, 1.22)	1.12 (8.14)	0.04	1.03
Injury, poisoning and procedural complications	552	0.54 (0.5, 0.59)	0.57 (197.87)	−0.93	0.52
Musculoskeletal and connective tissue disorders	394	0.82 (0.74, 0.9)	0.83 (15.28)	−0.42	0.75
Infections and infestations	330	0.67 (0.6, 0.74)	0.68 (53.1)	−0.72	0.61
Metabolism and nutrition disorders	325	1.65 (1.48, 1.84)	1.63 (79.89)	0.54	1.45
Skin and subcutaneous tissue disorders	316	0.62 (0.55, 0.69)	0.63 (71.56)	−0.83	0.56
Cardiac disorders	293	1.22 (1.09, 1.37)	1.21 (11.37)	0.11	1.08
Vascular disorders	131	0.66 (0.56, 0.79)	0.67 (22.24)	−0.83	0.56
Neoplasms benign, malignant and unspecified (incl cysts and polyps)	89	0.36 (0.3, 0.45)	0.37 (98.15)	−1.73	0.3
Eye disorders	74	0.4 (0.32, 0.5)	0.4 (67.25)	−1.64	0.32
Renal and urinary disorders	72	0.41 (0.32, 0.51)	0.41 (61.8)	−1.61	0.33
Social circumstances	39	0.9 (0.66, 1.24)	0.9 (0.41)	−0.6	0.66
Blood and lymphatic system disorders	36	0.23 (0.16, 0.32)	0.23 (93.87)	−2.56	0.17
Immune system disorders	36	0.35 (0.25, 0.49)	0.35 (43.12)	−1.95	0.25
Reproductive system and breast disorders	32	0.39 (0.28, 0.55)	0.39 (30.43)	−1.83	0.28
Surgical and medical procedures	32	0.25 (0.18, 0.36)	0.26 (70.57)	−2.44	0.18
Hepatobiliary disorders	30	0.35 (0.25, 0.5)	0.35 (35.87)	−1.99	0.25
Ear and labyrinth disorders	27	0.67 (0.46, 0.98)	0.67 (4.26)	−1.1	0.46
Product issues	21	0.14 (0.09, 0.21)	0.14 (114.08)	−3.41	0.09
Endocrine disorders	7	0.3 (0.14, 0.62)	0.3 (11.67)	−2.64	0.14
Congenital, familial and genetic disorders	1	0.04 (0.01, 0.26)	0.04 (25.82)	−5.88	0.01

Abbreviations: SOC, System Organ Class; ROR, Reporting Odds Ratio; PRR, Proportional Reporting Ratio; CI, Confidence Interval; IC025, lower 95% credibility limit of Information Component; EBGM05, Empirical Bayes Geometric Mean, lower 5% confidence limit.

**TABLE 7:** Distribution of positive signals for roflumilast adverse events at the SOC level.

soc_name_en	Number	Proportion
Psychiatric disorders	13	25.00%
Respiratory, thoracic and mediastinal disorders	8	15.38%
Investigations	6	11.54%
General disorders and administration site conditions	5	9.62%
Gastrointestinal disorders	4	7.69%
Nervous system disorders	4	7.69%
Cardiac disorders	3	5.77%
Metabolism and nutrition disorders	3	5.77%
Injury, poisoning and procedural complications	2	3.85%
Infections and infestations	1	1.92%
Musculoskeletal and connective tissue disorders	1	1.92%
Neoplasms benign, malignant and unspecified (incl cysts and polyps)	1	1.92%
Social circumstances	1	1.92%

The number of categories refers to the quantity of signals detected through statistical methods under the PT names classified by the SOC. Note that the number here refers to the count of PT categories, not the frequency of PT occurrences. Abbreviations: PT, Preferred Term; ROR, Reporting Odds Ratio; PRR, Proportional Reporting Ratio; CI, Confidence Interval; IC025, lower 95% credibility limit of Information Component; EBGM05, Empirical Bayes Geometric Mean, lower 5% confidence limit.

### 3.4 Disproportionality signal detection

Using four complementary disproportionality algorithms (ROR, PRR, IC, and EBGM) and applying established thresholds, 30 PTs were identified as significant safety signals for Roflumilast. These signals encompassed both well-recognized adverse effects and potential novel associations. The most frequently reported signals included Death (497 reports; ROR = 4.06, 95% CI 3.70–4.44), Diarrhoea (461; ROR = 5.09), Weight decreased (335; ROR = 8.29), Nausea (327; ROR = 2.86), Dyspnoea (286; ROR = 3.46), Insomnia (264; ROR = 6.71), Headache (238; ROR = 2.58), and Decreased appetite (222; ROR = 6.65) (Table 8). In addition, Chronic obstructive pulmonary disease (196 cases; ROR = 25.72) and Dizziness (189; ROR = 2.57) were common signals.

**TABLE 8:** The top 30 preferred terms ranked by frequency of roflumilast positive signals.

	PT	Number	ROR (95%CI)	PRR (X2)	IC025/IC-2SD	EBGM05
1	Death	497	4.06 (3.7, 4.44)	3.89 (1,081.86)	1.82	3.55
2	Diarrhoea	461	5.09 (4.63, 5.59)	4.89 (1,438.1)	2.14	4.45
3	Weight decreased	335	8.29 (7.43, 9.25)	8.03 (2067.28)	2.81	7.19
4	Nausea	327	2.86 (2.56, 3.19)	2.79 (380.74)	1.31	2.5
5	Dyspnoea	286	3.46 (3.08, 3.9)	3.39 (485.55)	1.57	3.01
6	Insomnia	264	6.71 (5.94, 7.58)	6.55 (1,244.41)	2.5	5.79
7	Headache	238	2.58 (2.27, 2.94)	2.54 (224.77)	1.15	2.23
8	Decreased appetite	222	6.65 (5.82, 7.6)	6.52 (1,039.7)	2.47	5.7
9	Chronic obstructive pulmonary disease	196	25.72 (22.32, 29.63)	25.19 (4,538.51)	4.27	21.78
10	Dizziness	189	2.57 (2.23, 2.97)	2.54 (177.82)	1.12	2.2
11	Tremor	135	5.43 (4.58, 6.43)	5.36 (479.88)	2.13	4.52
12	Back pain	126	3.63 (3.05, 4.33)	3.6 (237.22)	1.56	3.02
13	Anxiety	122	2.85 (2.38, 3.4)	2.82 (144.18)	1.21	2.36
14	Suicidal ideation	113	8.27 (6.87, 9.95)	8.18 (712.03)	2.67	6.78
15	Depression	96	2.77 (2.26, 3.38)	2.75 (107.11)	1.14	2.25
16	Influenza like illness	60	4.77 (3.7, 6.15)	4.75 (177.6)	1.79	3.68
17	Heart rate increased	46	3.12 (2.34, 4.17)	3.11 (66.05)	1.15	2.33
18	Intentional product misuse	46	3.57 (2.67, 4.77)	3.56 (84.59)	1.33	2.66
19	Nervousness	40	4.95 (3.63, 6.75)	4.93 (125.31)	1.72	3.61
20	Sleep disorder	31	3.05 (2.15, 4.35)	3.05 (42.67)	1.01	2.14
21	Medication error	26	3.17 (2.15, 4.65)	3.16 (38.4)	0.99	2.15
22	Restlessness	24	4.33 (2.9, 6.47)	4.32 (61.29)	1.35	2.89
23	Panic attack	23	4.19 (2.78, 6.31)	4.18 (55.71)	1.29	2.78
24	Nightmare	19	3.6 (2.29, 5.65)	3.59 (35.58)	1.02	2.29
25	Feeling jittery	17	5.84 (3.63, 9.4)	5.83 (68.04)	1.52	3.62
26	Frequent bowel movements	17	4.44 (2.76, 7.14)	4.43 (45.11)	1.21	2.75
27	Mood altered	17	4.19 (2.6, 6.74)	4.18 (41.19)	1.15	2.6
28	Abnormal dreams	16	3.7 (2.27, 6.05)	3.7 (31.5)	0.97	2.26
29	Emphysema	14	9.05 (5.36, 15.3)	9.04 (100)	1.81	5.34
30	Eating disorder	12	3.72 (2.11, 6.55)	3.71 (23.8)	0.82	2.11

Abbreviations: PT, Preferred Term; ROR, Reporting Odds Ratio; PRR, Proportional Reporting Ratio; CI, Confidence Interval; IC025, lower 95% credibility limit of Information Component; EBGM05, Empirical Bayes Geometric Mean, lower 5% confidence limit.

A notable cluster of neuropsychiatric signals was detected, including Suicidal ideation (113 cases; ROR = 8.27), Depression (96; ROR = 2.77), Anxiety (122; ROR = 2.85), as well as Nervousness, Panic attack, Nightmare, Restlessness, and Abnormal dreams (Table 8). Collectively, these reinforce concerns regarding psychiatric safety and support vigilance in patients with pre-existing psychiatric conditions.

Signals with the strongest disproportionality (highest ROR values) were often rare but striking: for example, Gastroduodenal ulcer (3 cases; ROR = 47.12) and Spirometry abnormal (3; ROR = 43.64) (Table 9). Among more common events, particularly high signal strength was observed for COPD (ROR = 25.72), Weight decreased (ROR = 8.29), Suicidal ideation (ROR = 8.27), Insomnia (ROR = 6.71), and Decreased appetite (ROR = 6.65). Several respiratory-related PTs (e.g., Increased bronchial secretion, Bronchial secretion retention, Sputum increased) also met signal criteria, whereas cardiovascular signals were sparse, with the exception of a few arrhythmia terms (e.g., Tachyarrhythmia, 4 cases; ROR = 10.48).

**TABLE 9:** The top 30 preferred terms ranked by Roflumilast positive signal strength.

	PT	Number	ROR (95%CI)	PRR (X2)	IC025/IC-2SD	EBGM05
1	Chronic obstructive pulmonary disease	196	25.72 (22.32, 29.63)	25.19 (4,538.51)	4.27	21.78
2	Gastroduodenal ulcer	3	47.12 (15.13,146.76)	47.1 (134.34)	0.46	15.01
3	Spirometry abnormal	3	43.64 (14.02,135.89)	43.63 (124.06)	0.45	13.91
4	Lung carcinoma cell type unspecified recurrent	3	33.39 (10.73,103.86)	33.38 (93.71)	0.43	10.67
5	Increased upper airway secretion	11	13.61 (7.53, 24.6)	13.59 (128.05)	1.89	7.5
6	Weight decreased	335	8.29 (7.43, 9.25)	8.03 (2067.28)	2.81	7.19
7	Suicidal ideation	113	8.27 (6.87, 9.95)	8.18 (712.03)	2.67	6.78
8	Increased bronchial secretion	6	15.26 (6.85, 34.01)	15.25 (79.7)	1.23	6.83
9	Insomnia	264	6.71 (5.94, 7.58)	6.55 (1,244.41)	2.5	5.79
10	Essential tremor	3	18.33 (5.9, 56.94)	18.32 (48.99)	0.33	5.88
11	Decreased appetite	222	6.65 (5.82, 7.6)	6.52 (1,039.7)	2.47	5.7
12	Bronchial secretion retention	3	17.07 (5.49, 53.01)	17.06 (45.23)	0.32	5.48
13	Emphysema	14	9.05 (5.36, 15.3)	9.04 (100)	1.81	5.34
14	Sputum increased	4	13.59 (5.09, 36.25)	13.58 (46.51)	0.66	5.08
15	Diarrhoea	461	5.09 (4.63, 5.59)	4.89 (1,438.1)	2.14	4.45
16	Pneumonia pseudomonal	3	14.36 (4.63, 44.61)	14.36 (37.2)	0.28	4.61
17	Tremor	135	5.43 (4.58, 6.43)	5.36 (479.88)	2.13	4.52
18	Food aversion	3	13.09 (4.22, 40.65)	13.09 (33.42)	0.26	4.21
19	Total lung capacity decreased	3	12.96 (4.17, 40.25)	12.96 (33.04)	0.25	4.17
20	Tachyarrhythmia	4	10.48 (3.93, 27.94)	10.47 (34.21)	0.56	3.92
21	Death	497	4.06 (3.7, 4.44)	3.89 (1,081.86)	1.82	3.55
22	Influenza like illness	60	4.77 (3.7, 6.15)	4.75 (177.6)	1.79	3.68
23	Nervousness	40	4.95 (3.63, 6.75)	4.93 (125.31)	1.72	3.61
24	Feeling jittery	17	5.84 (3.63, 9.4)	5.83 (68.04)	1.52	3.62
25	Body height decreased	11	6.49 (3.59, 11.73)	6.49 (51.01)	1.32	3.59
26	Dyspnoea	286	3.46 (3.08, 3.9)	3.39 (485.55)	1.57	3.01
27	Atrial tachycardia	3	9.52 (3.07, 29.54)	9.51 (22.82)	0.16	3.06
28	Back pain	126	3.63 (3.05, 4.33)	3.6 (237.22)	1.56	3.02
29	Inability to afford medication	8	5.95 (2.97, 11.91)	5.95 (32.89)	0.98	2.97
30	Restlessness	24	4.33 (2.9, 6.47)	4.32 (61.29)	1.35	2.89

Abbreviations: PT, Preferred Term; ROR, Reporting Odds Ratio; PRR, Proportional Reporting Ratio; CI, Confidence Interval; IC025, lower 95% credibility limit of Information Component; EBGM05, Empirical Bayes Geometric Mean, lower 5% confidence limit.

Overall, the concordance of all four disproportionality metrics lends robustness to these findings. The signal profile emphasizes Roflumilast’s established gastrointestinal intolerance and weight loss, while drawing attention to potentially serious neuropsychiatric and respiratory events that warrant ongoing pharmacovigilance.

## 4 Discussion

This pharmacovigilance study provides the most comprehensive real-world safety assessment of Roflumilast utilizing FAERS data. Our results confirm several well-documented adverse effects and identify potential new safety signals of clinical significance.

Gastrointestinal and metabolic events emerged as the most prominent safety signals. Diarrhea, nausea, and decreased appetite—together with significant weight loss—were among the most frequent and consistent findings. These observations mirror those from pivotal randomized controlled trials of roflumilast (Calverley et al., 2009; Fabbri et al., 2009), which reported increased risks of diarrhea (RR ≈ 2.95) and weight loss (RR ≈ 3.81), leading to higher discontinuation rates compared with placebo (Calverley et al., 2009; Wedzicha et al., 2016; Cilli et al., 2019). The observed rapid time-to-onset (median 4 days) also mirrors clinical experience that Gastrointestinal (GI) AEs often appear early. Mechanistically, Roflumilast’s increase in cAMP may activate gut pathways (e.g., GLP-1 signaling) that precipitate GI intolerance and anorexia (Wedzicha et al., 2016). Therefore, most events occurred within the first month of treatment, underscoring the need for close early monitoring.

Insomnia (264 cases, ROR≈6.7) and nervous-system events like headache were expected; notably, however, we also saw strong signals for depression and suicidal ideation. Suicidal ideation (113 cases, ROR≈8.3) and anxiety (122, ROR≈2.8) reached significance. These findings echo clinical warnings: both the FDA advisory review and post-marketing experience have cautioned that Roflumilast can aggravate psychiatric conditions (Ali, 2013). Earlier pooled analyses of clinical trials noted a small but higher incidence of suicide-related events on Roflumilast (≈0.08%) *versus* placebo (Lone and Oba, 2013). Similarly, FDA labeling prominently features warnings about psychiatric adverse effects, reflecting that an FDA panel initially postponed approval due to ‘significant side effects, primarily gastrointestinal and psychiatric issues, including suicidal behavior (Gupta, 2012). Our FAERS results underscore the necessity for clinicians to closely monitor mood and mental status, especially during the initial stages of treatment. The elevated ROR for insomnia and related ‘mood-altered’ events indicates that central nervous system PDE4 inhibition may be responsible for these findings.

Additionally, the respiratory/thoracic signal group warrants attention. Dyspnea (286 reports, ROR ≈3.5) and associated signals such as increased bronchial secretion were noted, along with several broad ‘lung’ terms (e.g., COPD coded as an event with ROR ≈25.7). These observations are interpreted cautiously: many Roflumilast patients suffer from severe COPD, leading to frequent coding of respiratory symptoms or chronic COPD as adverse events. For instance, ‘COPD’ itself had one of the highest RORs, which likely reflects the treated condition rather than a direct adverse effect. Similarly, respiratory infections were not significantly enriched in our analysis; rates of pneumonia and other infections with Roflumilast have been comparable to those observed with placebo in clinical trials (Naseem et al., 2022), and FAERS data revealed only sporadic signals (e.g., few ‘pneumonia pseudomonal’ cases).

Cardiovascular adverse events were infrequent in the FAERS, which is reassuring considering the baseline risk in COPD patients. Notably, a recent pooled safety meta-analysis revealed no increase in major cardiovascular events associated with Roflumilast, and even indicated a lower rate of major adverse cardiac events (MACE) compared to placebo (White et al., 2013). Our data did not reveal any strong cardiac signals aside from a few arrhythmia terms (e.g., atrial tachycardia, which had very few cases). Atrial fibrillation incidence was slightly higher in one meta-analysis (Oba and Lone, 2013), but this did not manifest as a clear signal, potentially due to underreporting or confounding by COPD severity (Wedzicha et al., 2016).

Notably, certain rare but high-strength signals, such as gastroduodenal ulcers and spirometry abnormalities, were identified. Although the number of cases was limited, the degree of disproportionality necessitates careful interpretation: these findings could either indicate rare, true drug-related events or reporting artifacts. Further validation in larger datasets, such as EudraVigilance or WHO VigiBase, is warranted.

Roflumilast’s adverse profile is linked to its pharmacological action. By inhibiting PDE4 in both the central nervous system (CNS) and the gastrointestinal tract, it enhances neuronal and gastrointestinal cyclic adenosine monophosphate (cAMP) signaling (Li et al., 2018). This approach may decrease appetite and enhance GLP-1 levels, which promotes weight loss. However, it can also disrupt central neurotransmission, potentially resulting in insomnia, anxiety, and, in rare cases, suicidal ideation (Vollert et al., 2012). GI effects are a well-documented characteristic of this class (similar to those observed with other PDE4 inhibitors, such as cilomilast) (Li et al., 2018). Notably, approximately threefold more potent than its active metabolite roflumilast N-oxide, which is highly abundant in plasma. Nevertheless, current pharmacokinetic data indicate limited CNS penetration of both compounds, suggesting that neuropsychiatric adverse events are more likely related to indirect mechanisms rather than direct central exposure (Hojo et al., 2016; Li et al., 2017).

This study, conducted as a spontaneous-reporting analysis, inherently faces certain limitations. FAERS data are susceptible to underreporting, reporting biases, and varying data quality. It is important to note that disproportionality signals do not establish causality or provide incidence quantification (Cutroneo et al., 2024). The signals for death and COPD likely reflect underlying disease severity and reporting practices rather than drug toxicity. The small counts for certain signals, such as gastroduodenal ulcers and increased bronchial secretions, result in wide confidence intervals and potential false positives. Conversely, frequently occurring yet nonspecific events may be overlooked if they are also common with other medications. Importantly, patient-level confounders, such as smoking history and concurrent medications like steroids or bronchodilators, cannot be controlled in the FAERS database. Consequently, these findings are primarily hypothesis-generating. However, they inform vigilance: for example, our findings suggest that despite clinical trials showing no significant infection or seizure risk, clinicians should monitor for severe adverse outcomes, particularly in patients with psychiatric histories. Future research should encompass pharmacoepidemiologic studies, such as cohort analyses of claims or electronic health record (EHR) data, to estimate absolute risk and identify predisposing factors for Roflumilast AEs. Furthermore, additional pharmacovigilance efforts, including updates to the FAERS, EudraVigilance, and the World Health Organization’s VigiBase, are necessary to validate these signals and monitor emerging trends.

## 5 Conclusion

In summary, the FAERS analysis confirms Roflumilast’s established safety profile, characterized by high rates of gastrointestinal upset, weight loss, and insomnia, while also highlighting concerning neuropsychiatric signals. The majority of adverse events associated with Roflumilast occurred early in treatment, with a median onset of 4 days and 72% occurring within 30 days. This underscores the critical need for close monitoring during the initial weeks of therapy. Our findings emphasize the importance of counseling patients regarding gastrointestinal side effects and weight loss, and recommend screening for mood disturbances both before and during treatment. The four-method signal detection approach effectively captures both expected and unexpected events, enhancing pharmacovigilance and patient education. Clinicians must promptly identify and manage early adverse symptoms, such as treating nausea aggressively or adjusting doses and discontinuing treatment if psychiatric symptoms arise. This analysis highlights the balance between Roflumilast’s benefits in reducing COPD exacerbations and its tolerability. Early adverse event monitoring and timely interventions are crucial for patient safety. Caution is advised for patients with a history of depression or suicidal ideation. These findings can guide prescribing practices and inform future safety studies as Roflumilast usage expands.

## Data Availability

The original contributions presented in the study are included in the article/Supplementary Material, further inquiries can be directed to the corresponding authors.

## Supplementary material

SUPPLEMENTARY TABLE S1 presents the full disproportionality analysis results at the Preferred Term (PT) level for the target drug.

SUPPLEMENTARY TABLE S2 includes System Organ Class (SOC)-level analyses, covering (i) SOC-level signal results, (ii) adverse reaction events affecting SOCs, and (iii) the number of SOCs involved in adverse reaction events.

SUPPLEMENTARY TABLE S3 shows the distribution of risk signals across organ system categories, including (i) organ system categories involved in risk signals and (ii) the number of organ systems involved.

SUPPLEMENTARY TABLE S4 provides comprehensive summaries of drug-related risk signals, including (i) target drug risk signal results and (ii) the distribution of organ systems involved in adverse reaction events under positive signals.
